# *Candida albicans* stimulates *Streptococcus mutans* microcolony development via cross-kingdom biofilm-derived metabolites

**DOI:** 10.1038/srep41332

**Published:** 2017-01-30

**Authors:** Dongyeop Kim, Arjun Sengupta, Tagbo H. R. Niepa, Byung-Hoo Lee, Aalim Weljie, Veronica S. Freitas-Blanco, Ramiro M. Murata, Kathleen J. Stebe, Daeyeon Lee, Hyun Koo

**Affiliations:** 1 Biofilm Research Laboratory, Department of Orthodontics and Divisions of Pediatric Dentistry & Community Oral Health, School of Dental Medicine, University of Pennsylvania, Philadelphia, PA, USA; 2 Department of Systems Pharmacology & Translational Therapeutics, University of Pennsylvania, Philadelphia, PA, USA; 3 Department of Chemical and Biomolecular Engineering, University of Pennsylvania, Philadelphia, PA, USA; 4 Department of Food Science, Gachon University, Seongnam, South Korea; 5 Herman Ostrow School of Dentistry, University of Southern California, Los Angeles, CA, USA

## Abstract

*Candida albicans* is frequently detected with heavy infection of *Streptococcus mutans* in plaque-biofilms from children affected with early-childhood caries, a prevalent and costly oral disease. The presence of *C. albicans* enhances *S. mutans* growth within biofilms, yet the chemical interactions associated with bacterial accumulation remain unclear. Thus, this study was conducted to investigate how microbial products from this cross-kingdom association modulate *S. mutans* build-up in biofilms. Our data revealed that bacterial-fungal derived conditioned medium (BF-CM) significantly increased the growth of *S. mutans* and altered biofilm 3D-architecture in a dose-dependent manner, resulting in enlarged and densely packed bacterial cell-clusters (microcolonies). Intriguingly, BF-CM induced *S. mutans gtfBC* expression (responsible for Gtf exoenzymes production), enhancing Gtf activity essential for microcolony development. Using a recently developed nanoculture system, the data demonstrated simultaneous microcolony growth and *gtfB* activation *in situ* by BF-CM. Further metabolites/chromatographic analyses of BF-CM revealed elevated amounts of formate and the presence of *Candida*-derived farnesol, which is commonly known to exhibit antibacterial activity. Unexpectedly, at the levels detected (25–50 μM), farnesol enhanced *S. mutans*-biofilm cell growth, microcolony development, and Gtf activity akin to BF-CM bioactivity. Altogether, the data provide new insights on how extracellular microbial products from cross-kingdom interactions stimulate the accumulation of a bacterial pathogen within biofilms.

Early childhood caries (ECC) is a highly prevalent and difficult to treat biofilm-dependent oral disease, afflicting mostly underprivileged children worldwide, resulting in an estimated annual expenditures of >$120 billion in the US alone^1^,^2^. Children affected with ECC display heavy infection with *Streptococcus mutans* accompanied by protracted feeding of dietary sugars, such as sucrose^3^,^4^,^5^, leading to rapid accumulation of virulent biofilms that cause rampant destruction of the teeth^1^,^6^.

Caries-causing biofilms develop when bacteria interact with dietary sugars and accumulate on tooth surface, forming densely packed cell clusters (or microcolonies) that are firmly adherent and enmeshed in an extracellular matrix of polymeric substances such as exopolysaccharides (EPS)^7^. EPS, particularly glucans, enhance bacterial adhesion and cohesion, while forming a diffusion-limiting matrix that protects the embedded bacteria and helps to acidify the local microenvironment. These biofilm properties promote the growth of an acidogenic microbiota, and eventually lead to the onset of dental caries^8^,^9^,^10^.

*S. mutans* is regarded as one of the key etiologic agents of ECC because this pathogen can efficiently catalyze dietary sucrose into extracellular glucans using several exoenzymes (glucosyltransferases or Gtfs) making it a primary EPS producer in the oral cavity, while being both acidogenic and acid-tolerant^7^. However, *S. mutans* may not act alone in cariogenic biofilms, as additional organisms may be involved^6^. Results from clinical studies reveal that *C. albicans* is frequently detected with high numbers of *S. mutans* in plaque-biofilms from children with ECC^11^,^12^,^13^,^14^,^15^. These findings are intriguing, because this opportunistic fungus usually neither binds well with *S. mutans,* nor colonizes teeth effectively on its own^16^,^17^,^18^. Rather, *C. albicans* interacts with commensal (viridans) streptococci and form biofilms on acrylic/mucosal surfaces^19^,^20^ to cause oral mucosal infections^21^,^22^. However, physical coadhesion of *S. mutans* and *C. albicans* is drastically enhanced in the presence of sucrose; these conditions also promote biofilm formation^17^,^23^,^24^,^25^.

Further *in vitro* studies have demonstrated that *S. mutans*-derived Gtfs play a key role by binding avidly to the fungal surface in an enzymatically active form^17^,^26^. When sucrose is available, surface-bound exoenzymes produce large amounts of EPS on the fungal surface, which provide enhanced binding sites for *S. mutans,* thereby promoting their adhesive interactions and crosskingdom biofilm development^25^. Using a rodent model of the disease, an enhancement of *S. mutans* levels in plaque-biofilms was observed when co-infected with *C. albicans* and exposed to a sucrose-rich diet^25^. Importantly, the virulence was significantly increased, leading to the onset of rampant caries on teeth similar to those found in ECC.

Associations between bacteria and fungi can be antagonistic or cooperative^22^,^27^,^28^. Once together within biofilms, these organisms may cooperate with each other for provision of substrates/metabolites or growth stimulating factors when conditions are conducive for ECC. For example, *Candida* does not metabolize sucrose efficiently^29^, and could benefit from cross-feeding of sucrose break-down products (glucose and fructose) by *S. mutans*^30^,^31^. Conversely, the presence of *C. albicans* dramatically modifies the physical environment of the biofilms by increasing EPS production that is critical for *S. mutans* accumulation and formation of microcolonies^25^. Furthermore, *C. albicans* appears to activate *S. mutans* genes associated with EPS/fitness^25^ and competence genes^31^. However, the manner in which such chemical interactions and secreted molecules stimulate *S. mutans* growth and accumulation remains unclear.

Thus, this study investigates whether extracellular microbial products derived from *S. mutans*-*C. albicans* biofilm interactions modulate the bacterial population build-up within biofilms. Our data revealed that bacterial-fungal conditioned medium (BF-CM) significantly increased the growth of *S. mutans* biofilm cells and enhanced microcolony formation through triggering of Gtfs activity via up-regulation of *gtfBC*. Using a recently developed microcapsule-based nanoculture and a *S. mutans gtfB*::green fluorescence protein promoter, the data demonstrated that BF-CM stimulated bacterial cells confined within these semipermeable chambers to develop into microcolonies while inducing *gtfB* expression *in situ*. Metabolite profiling and chromatographic analyses of BF-CM revealed elevated amounts of formate and the presence of farnesol, a quorum-sensing molecule from *C. albicans* that is commonly understood to exhibit antibacterial activity. Surprisingly, farnesol levels (25–50 μM) detected in BF-CM enhanced *S. mutans* cell growth, microcolony development, and Gtfs activity in a manner similar to that observed with BF-CM. However, higher concentrations (>100 μM) of farnesol inhibited *S. mutans* growth. Thus, farnesol is a potential key modulator in this crosskingdom interaction, and *S. mutans* growth responds non-monotonically to farnesol concentration. Altogether, this study provides new insights on the chemical interactions between an opportunistic fungus (*C. albicans*) and an oral pathogen (*S. mutans*), which could help explain how their association lead to virulent biofilms in ECC.

## Results

### Bacterial-fungal derived conditioned medium (BF-CM) promotes *S. mutans* growth and microcolony development

**Figure 1:**
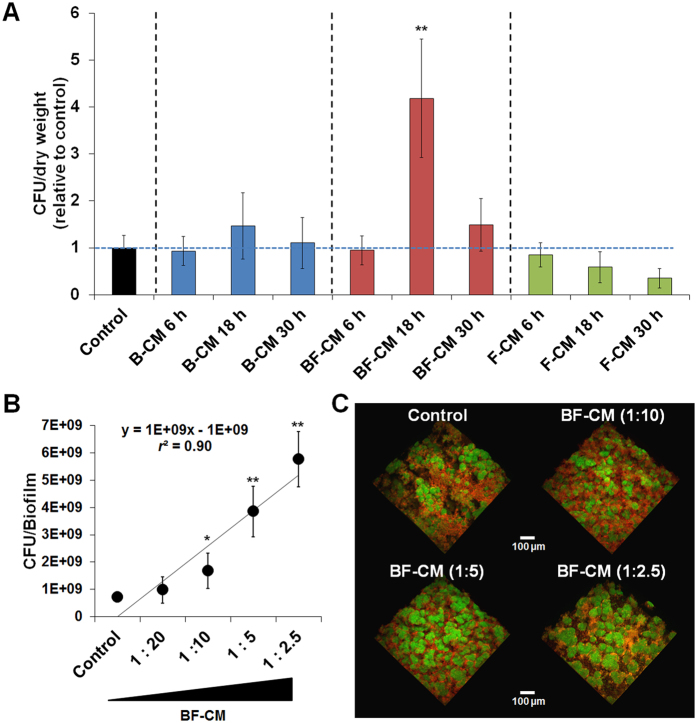
Influences of conditioned medium on the growth of *S. mutans* biofilm cells and microcolony development. (**A**) Conditioned medium (CM) was collected and prepared from single-species bacterial (B-CM), fungal (F-CM) or bacterial-fungal (BF-CM) biofilms at different time points. *S. mutans* biofilms were grown on saliva-coated hydroxyapatite (sHA) disc surface in each of the CM preparations (1:5 vol/vol, CM:UFTYE). The viable cells number (colony forming units (CFU)/biofilm) was normalized by dry weight (mg) (n = 8). (**B**) Dose-response effects of BF-CM on the growth of *S. mutans* biofilm cells (n = 4). (**C**) Representative 3D rendering images of biofilms formed with different dilutions of BF-CM and analyzed via multi-photon confocal laser scanning microscopy. *S. mutans* cells stained with SYTO 9 are depicted in green, while EPS labelled with Alexa Fluor 647 is shown in red. Data represent mean ± SD. The quantitative data were subjected to analysis of variance (ANOVA) in the Tukey’s HSD test for a multiple comparison. Values are significantly different from each other at *P < 0.05 or **P < 0.01.

Conditioned medium (CM) was collected and prepared from single-species bacterial (*S. mutans*; B-CM), single-species fungal (*C. albicans*; F-CM) or bacterial-fungal (BF-CM) biofilms at three time-points (6, 18 and 30 h) corresponding to active biofilm formation using our sHA model (Figs 1A and S1; also Fig. S2 shows their overall architecture). Initially the influences of various CM preparations on *S. mutans* biofilm-cells growth were evaluated. The results showed that BF-CM collected at 18 h significantly promoted bacterial accumulation within biofilms compared to control (no supplementation, *P* < 0.001) (Fig. 1A). In contrast, no significant increase in the bacterial cell population was detected when *S. mutans* was grown in CM preparations from single-species biofilms or from BF-CM at other time-points. It appears that during the initial phase of active biofilm formation (between 6 h to 18 h) the presence of cross-kingdom metabolites could be enhancing *S. mutans* cell growth, while at the later time point (30 h) this effect is attenuated possibly due to reduced microbial/metabolic activity. Thus, hereafter further experiments were focused on the bioactivity of BF-CM collected at 18 h on biofilm formation by *S. mutans*. Using different dilutions of BF-CM, the data showed a clear trend of increasing number of viable *S. mutans-*biofilm cells (>6-fold increase at highest BF-CM content vs. no supplementation) in a dose-dependent manner (*r*^2^ = 0.9; Fig. 1B) suggesting that BF-CM contains bacterial growth-inducing factors.

**Figure 2:**
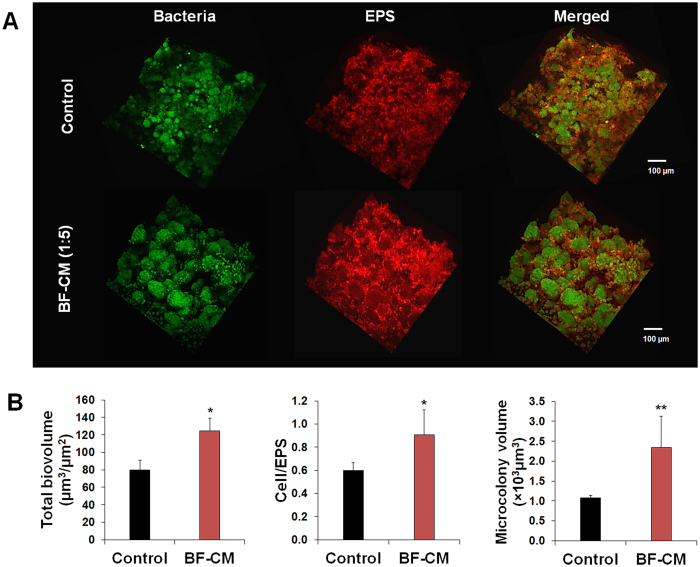
Three-dimensional (3D) architecture and quantitative computational analysis of biofilm formed in BF-CM. (**A**) Representative confocal images of *S. mutans* biofilms grown in BF-CM (1:5vol/vol, CM:UFTYE) and without supplementation (control). The bacterial microcolonies are depicted in green (SYTO 9), while the EPS-matrix is depicted in red (Alexa Fluor 647). (**B**) Quantitative analysis of total biovolume (biomass), cell/EPS ratio and microcolony volume (size) was performed using COMSTAT. Data represent mean ± SD (n = 8). A pairwise comparison between control and BF-CM was conducted using student’s *t*-test. Values are significantly different from each other at *P < 0.05 or **P < 0.01.

Furthermore, confocal imaging revealed significant alterations in the biofilm three-dimensional (3D) architecture with enhanced bacterial clustering and accumulation when grown in the presence of increased amounts of BF-CM (Fig. 1C). It showed enlarged and densely packed microcolonies (in green) enmeshed in a well-developed EPS matrix (in red) (Fig. 2A). Quantitative computational analyses (COMSTAT) showed significantly higher total biomass and cell/EPS ratio in *S. mutans* biofilms formed in BF-CM, while the volume of the microcolony was increased by ~2.5 fold (vs. control, *P* < 0.01) (Fig. 2B). Therefore, BF-CM appears to enhance microcolony development with an elevated carriage of *S. mutans* cells.

### BF-CM modulates the dynamics of *S. mutans*-derived glucosyltransferases (Gtfs) activity

**Figure 3:**
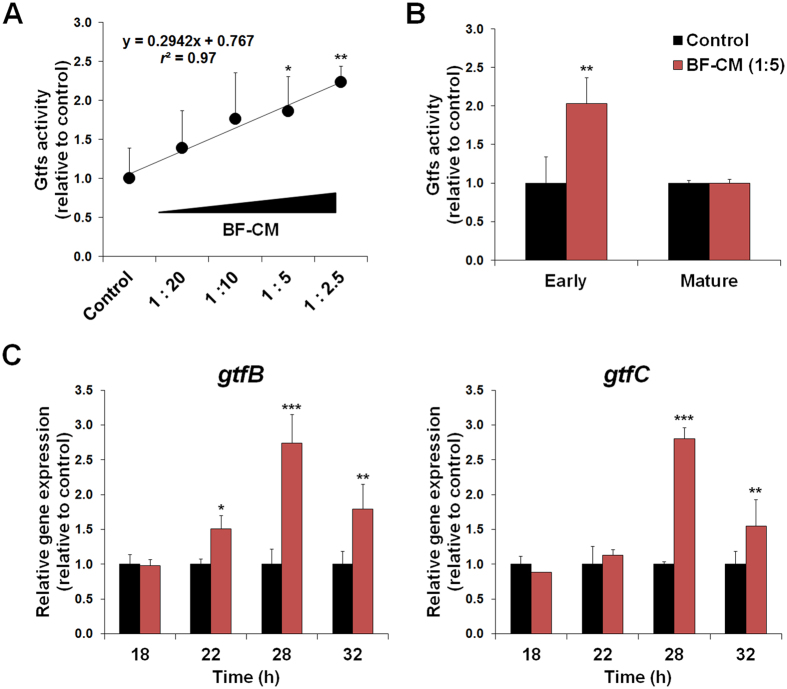
Dynamics of glucosyltransferases (Gtfs) activity and *gtfBC* expression profiles in biofilms formed in BF-CM. (**A**) Dose-response effects of BF-CM (diluted in UFTYE, vol/vol) on Gtfs activity. The data were subjected to linear regression analysis and analysis of variance (ANOVA) in the Tukey’s HSD test for a multiple comparison. Correlation coefficient between increasing amounts of BF-CM and Gtfs activity was *r*^2^ = 0.97. (**B**) Temporal effects of BF-CM on Gtfs activity at different stages of biofilm development; early-stage (18–28 h) and matured biofilms (28–42 h). (**C**) Influence of BF-CM (collected at 18 h) on *gtfB* and *gtfC* gene expression by *S. mutans* biofilms was analyzed at 18, 22, 28, and 32 h of development. A pairwise comparison between control and BF-CM was conducted using student’s *t*-test. Data represent relative ratio to control (defined as 1); mean ± SD (n = 4). Values are significantly different from each other at *P < 0.05, **P < 0.01 or ***P < 0.001.

Since microcolony development is linked with the activity of Gtf exoenzymes^32^,^33^, the impact of BF-CM on total Gtfs activity and *gtf* gene expression during *S. mutans* biofilm formation was further investigated (Fig. 3). The biofilms were formed with varying BF-CM dilutions, and the supernatant collected for Gtfs activity as measured by scintillation counting using radiolabeled sucrose. Linear regression analysis showed that Gtfs activity correlated strongly with increasing amounts of extracellular microbial products in BF-CM (*r*^2^ = 0.97) (Fig. 3A). Also, temporal effects were examined to confirm whether the inducing effects of BF-CM on Gtfs activity were dependent on the stage of biofilm development. Interestingly, Gtfs activity was significantly enhanced by BF-CM (~2-fold increase vs. control, *P* < 0.01; Fig. 3B) during the early-stage of biofilm formation (18–28 h). However, no significant effects were observed in matured biofilms (42 h) (Fig. 3B). Hence, exposure to BF-CM can increase Gtfs activity during biofilm initiation, promoting *S. mutans* ability to form cell clusters and microcolonies onto sHA surface.

Furthermore, additional experiments were conducted to examine the manner in which BF-CM influences the pattern of *gtfB* and *gtfC* expression, which encodes the primary Gtfs associated with microcolony development by *S. mutans*^33^. Notably, *gtfB* expression was up-regulated as early as 22 h within biofilms formed with BF-CM, while both *gtfB* and *gtfC* were dramatically induced (~3-fold increase vs. control, *P* < 0.001; Fig. 3C) at 28 h of development. Altogether, the data reveal that BF-CM contains microbial products that are capable of both stimulating bacterial growth and inducing Gtfs activity, consistent with elevated population of bacterial cells (Fig. 1) and development of enlarged microcolonies within biofilms formed in the presence of BF-CM (Fig. 2B).

### Microcolony assembly and *gtfB* expression *in situ* using a semipermeable nanoculture system

**Figure 4:**
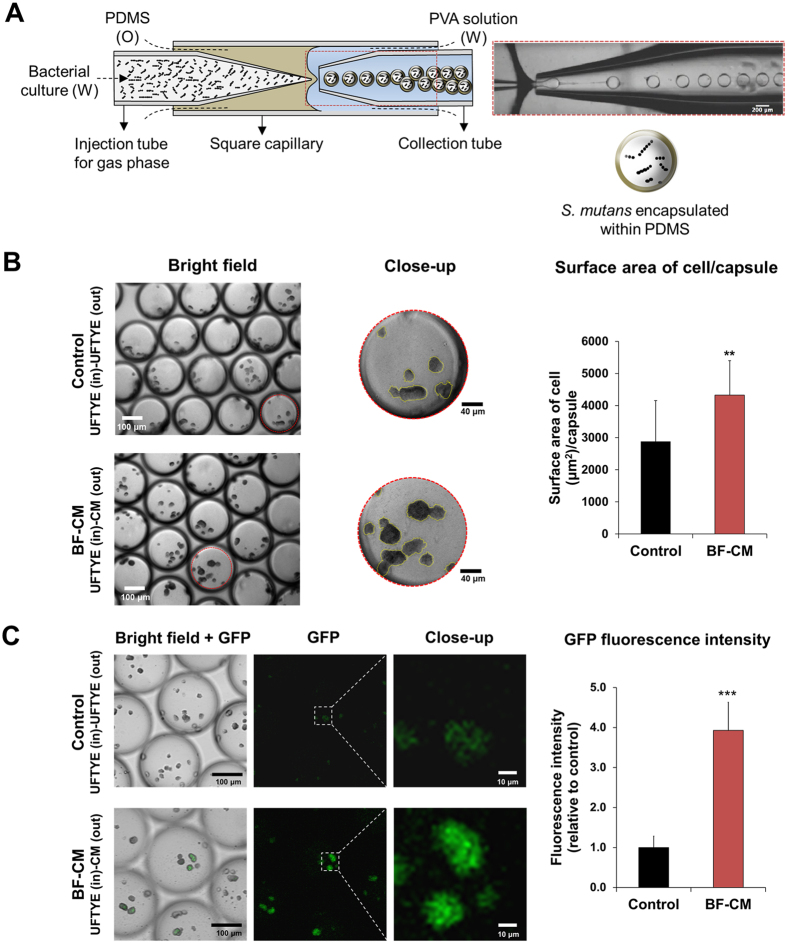
Microcolony assembly and *gtfB* expression *in situ* using a microfluidics-generated nanoculture system. (**A**) Defined cell population of *S. mutans* (~30 cells per nanoculture) was encapsulated in a semipermeable polydimethylsiloxane (PDMS)-based nanoculture (microcapsules). (**B**) *S. mutans* cells were inoculated in PDMS nanocultures with culture medium (UFTYE) and seeded in the medium (outside) containing either BF-CM (1:5 vol/vol, CM:UFTYE) or without supplementation (control). The surface area of cell occupying the interior of the capsule was measured by Image J. Data represent mean ± SD (n = 12). (**C**) Microcolony formation and *gtfB* expression via *S. mutans* strain expressing GFP under the control of *gtfB* promoter; P_*gtfB*_*::gfp* were determined using optical and confocal microscopy. The GFP fluorescent intensity was measured by using Image J. Data represent mean ± SD (n = 20). A pairwise comparison between control and BF-CM was conducted using student’s *t*-test. Values are significantly different from each other at **P < 0.01, ***P < 0.001.

To further demonstrate the biological properties of BF-CM, a newly developed nanoliter-scale culturing system was used, which allows microbial growth within spatially confined yet permeable microcapsules, mimicking biofilm microenvironments. Using a microfluidics fabrication method, defined cell population of *S. mutans* can be directly inoculated and encapsulated within a physical shell surrounding the bacterial culture (4 to 5 nanoliter) (Fig. 4A). The nanocultures confine microorganisms within a semipermeable polydimethylsiloxane (PDMS) membrane containing growth medium, while allowing transport of small molecules^34^. Since this platform allows chemical fluxes between internal and external environments, it was determined whether *S. mutans* cells grown within the nanoculture respond to bioactive molecules in the BF-CM placed outside of the microcapsules.

*S. mutans* cells were encapsulated in PDMS nanocultures with culture medium, and then seeded in the same medium (outside) containing BF-CM or without supplementation (control). Microcolony formation and *in situ gtfB* expression (using P_*gtfB*_::*gfp* strain) within the microcapsules were assessed via optical and confocal microscopy. Consistent with the findings using *de facto* biofilms, the microcolonies appeared visually larger in the nanocultures of *S. mutans* placed in BF-CM, while the surface area of cell occupying the interior of the capsule was significantly higher than control (*P* < 0.01; Fig. 4B). Future studies shall include quantitative measurements of the size (e.g. diameter and volume) of the microcolonies within each of the microcapsule. Furthermore, *gtfB* expression by these bacterial microcolonies was significantly enhanced, showing ~4-fold increase as determined via measurement of fluorescence intensity of green fluorescence protein (GFP) (vs. control, *P* < 0.001) (Fig. 4C). Together, these observations suggest that BF-CM harbors small molecules, which are capable of diffusing through the PDMS shell and induce the resident bacterial cells to promote microcolony growth with enhanced *gtfB* expression.

### Chemical characterization of conditioned medium

**Figure 5:**
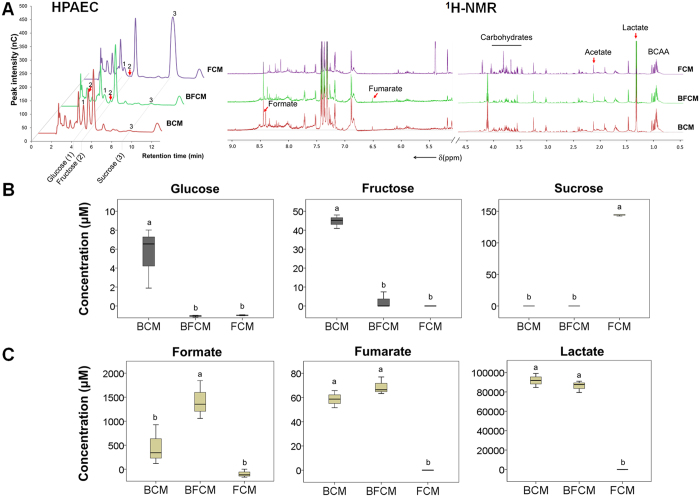
The composition of carbohydrates and metabolites in the conditioned medium (CM). CM was collected and prepared from single-species bacterial (B-CM), fungal (F-CM) or bacterial-fungal (BF-CM) biofilms as described in Materials and Methods. (**A**) HPAEC chromatograms of carbohydrate profile and ^1^H-NMR analysis for metabolites (organic acids shown). (**B**) The concentrations of glucose, fructose and sucrose as the main carbohydrates in undiluted CM. (**C**) The concentrations of formate, fumarate and lactate as the main metabolites in undiluted CM. Data represent mean ± SD (n = 3). The data were subjected to analysis of variance (ANOVA) in the Tukey’s HSD test for a multiple comparison. The letters (a and b) denote significate differences (*P* < 0.01).

Our data suggest that conditioned medium from bacterial-fungal biofilm interaction contains specific *S. mutans*-growth inducing and Gtfs-activating molecules that could be critical for enhanced bacterial accumulation observed in previous *in vitro* and *in vivo* studies^24^,^25^,^35^. Therefore, the composition of carbohydrates and metabolites in BF-CM as well as in the conditioned medium from single-species biofilms (B-CM and F-CM) was characterized using colorimetric, high-performance anion-exchange chromatography (HPAEC) and ^1^H nuclear magnetic resonance (^1^H-NMR) methods (Fig. 5A). Since *S. mutans* efficiently metabolizes sucrose^36^,^37^, this carbohydrate was almost completely utilized in the conditioned medium from single-species bacterial (B-CM) or bacterial-fungal (BF-CM) biofilms (Fig. 5B). In contrast, most of the carbohydrate remained in the medium from single-species fungal biofilm (F-CM), consistent with *C. albicans* inefficient utilization of sucrose^29^. Fructose derived from the breakdown of sucrose was detected at high concentration in *S. mutans* conditioned medium. However, there was a significant difference (14.7-fold, *P* < 0.01) between the concentration of fructose contained in B-CM (44 μM) and BF-CM (3 μM), indicating active fructose metabolism in *S. mutans-C. albicans* biofilms.

Changes in the metabolite composition and content in the conditioned medium may reflect chemical interactions between *S. mutans* and *C. albicans* within biofilms. Hence, the profile of extracellular metabolites (e.g. organic acids, alcohols, sugar alcohols, amino acids) in BF-CM, B-CM and F-CM was determined (Figs 5C and S3). The results of metabolite composition analyses revealed that lactate was the major metabolite detected in BF-CM and B-CM (Figs 5C and S3). Notably, the formate concentration was significantly increased in BF-CM (vs. others, *P* < 0.01).

**Figure 6:**
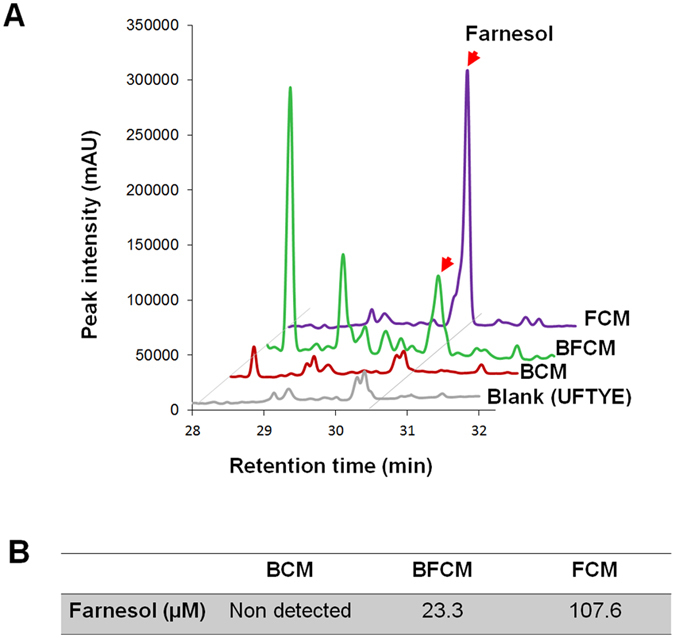
Quantification of farnesol in the conditioned medium (CM). (**A**) HPLC profile of farnesol was obtained from CM extracted with EtOAc. The peak at 30.5 min is of farnesol (red arrows). Conditioned medium (CM) was collected and prepared from single-species bacterial (B-CM), fungal (F-CM) or bacterial-fungal (BF-CM) biofilms at 18 h. (**B**) Biological concentration of farnesol detected in the CM.

After initial chemical characterization, further biological evaluation was conducted to determine whether the bacterial/microcolony-growth inducing factor could be a functional protein. Since heat treatment, digestion with proteinase K, and combination of both did not alter the biological properties of BF-CM but promoted *S. mutans* cell growth compared to non-treated BF-CM (*P* > 0.05) (Fig. S4), the extracellular microbial factors appeared to be non-proteinaceous or non-enzymatic molecules that were small and diffusible. In parallel, BF-CM (treated or non-treated, as described above) unexpectedly inhibited *C. albicans* morphogenesis, blocking the transition from yeast to hyphal forms in a dose-dependent manner (Fig. S5). Since farnesol is a small, diffusible quorum-sensing (QS) molecule secreted by *C. albicans,* and is well-known to inhibit the yeast-to-hyphae transition^38^, additional chromatographic analyses were performed to confirm whether farnesol could be detected in BF-CM (Fig. 6). BF-CM or F-CM contained detectable amounts of farnesol, with an estimated concentration of 23.3 and 107.6 μM, respectively based on HPLC quantification (Fig. 6). As expected, farnesol was undetected in B-CM since *S. mutans* cannot produce and secrete this molecule.

### Low concentrations of farnesol enhance the growth of *S. mutans* biofilm cells and Gtfs activity

**Figure 7:**
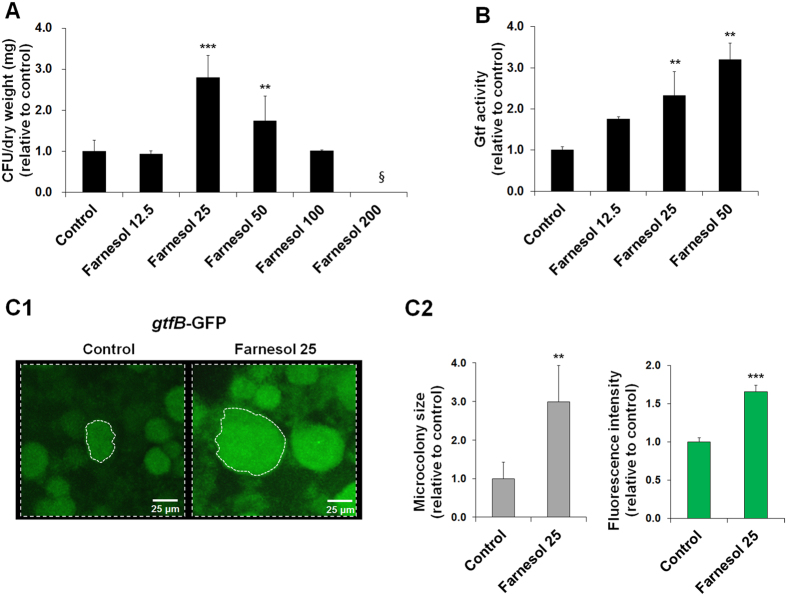
Influences of farnesol at levels found in BF-CM on Gtfs activity and *S. mutans* biofilm formation. (**A**) Influence of farnesol at different concentrations (0–200 μM) on the *S. mutans* biofilm cell growth. Data represent mean ± SD (n = 4). The data were subjected to analysis of variance (ANOVA) in the Tukey’s HSD test for a multiple comparison. ^§^Indicates non-detected. (**B**) Gtfs activity in *S. mutans* biofilms grown in the presence of farnesol at a range of concentrations (12.5, 25 and 50 μM). Data represent mean ± SD (n = 6). The data were subjected to analysis of variance (ANOVA) in the Tukey’s HSD test for a multiple comparison. (**C1**) Microcolony development and *gtfB* expression via P_*gtfB*_*::gfp S. mutans* in biofilms formed on sHA surface in the presence of farnesol at 25 μM. Fluorescence images were obtained using confocal laser scanning microscopy. (**C2**) The microcolony size and GFP fluorescence intensity were measured by COMSTAT and Image J. Data represent mean ± SD (n = 4). A pairwise comparison between control and farnesol was conducted using student’s *t*-test. Values are significantly different from each other at **P < 0.01 or ***P < 0.001.

The identification of farnesol in BF-CM is intriguing because this fungal signaling molecule also exhibits antibacterial activity against *S. mutans* biofilms at high concentrations (>1 mM)^39^. Thus, the biological effects of farnesol at a range of concentrations (12.5, 25 and 50 μM) akin to that found in BF-CM was investigated. To be consistent with the experiments using BF-CM, the influences of farnesol on both *S. mutans* biofilm formation and the Gtfs activity were examined. Surprisingly, the results revealed that farnesol at these low concentrations actually induced the growth of *S. mutans* biofilm cells (Fig. 7A), and concomitantly enhanced Gtfs activity in a dose-dependent manner (vs. control; Fig. 7B). In contrast, bacterial growth was inhibited by farnesol at high concentrations (>100 μM; Fig. 7A).

Since the amount of farnesol detected in BF-CM (~25 μM) promoted the growth of *S. mutans* and the Gtfs activity, further experiments were conducted to confirm whether farnesol can stimulate microcolony development and *gtfB* expression (via P_*gtfB*_::*gfp*). Strikingly, farnesol induced the formation of enlarged microcolonies with elevated expression of *gtfB* (Fig. 7C) in a manner similar to that observed with BF-CM. Together, our data suggest that farnesol may be an important molecule mediating this cross-kingdom interaction, with a potential role in regulating *S. mutans* accumulation and microcolony development within cariogenic biofilms.

## Discussion

Our findings reveal that extracellular factors derived from the association between *S. mutans* and *C. albicans* stimulates bacterial growth within the biofilms. Gtfs activity via up-regulation of *gtf* genes, particularly *gtfB*, is triggered by secreted fungal factors, which in turn enhances microcolony formation by *S. mutans*. The insoluble glucans produced by GtfB can provide bacterial binding sites for *S. mutans* accumulation, while serving as a structural scaffold that are essential for microcolony development^33^,^40^,^41^. The microcolonies also facilitate the formation of localized acidic microenvironments that promote acid-dissolution of the adjacent tooth enamel^10^. Interestingly, elevated GtfB levels in human saliva^42^ and increased amounts of insoluble glucans in plaque-biofilms^43^,^44^ have been associated with caries activity in ECC-affected children. Furthermore, *S. mutans* defective in *gtfB* is unable to form microcolonies^32^, to infect teeth or to cause severe carious lesions in rodent caries model^45^. Thus, it is plausible that the presence of *C. albicans* factors in plaque-biofilms might play an important role in *gtfB* activation *in situ,* and thereby in the enhanced *S. mutans* virulence and development of ECC *in vivo*, which can be further elucidated in future longitudinal clinical studies.

Interestingly, the biochemical characterization using a combination of heat treatment and digestion with proteinase K, and chromatographic analyses indicate that farnesol may be a key chemical modulator. The role of farnesol in mediating *C. albicans* yeast-to-hyphae transition while exhibiting antibacterial activity has long been appreciated^38^,^39^. However, *S. mutans* growth responds non-monotonically to farnesol concentration. At low farnesol levels (~25 μM) found in the conditioned medium from co-cultured biofilms, it enhances bacterial cell growth and triggers *gtfB* expression/activity within biofilms, which correlated with microcolony development by *S. mutans*. In contrast, high concentrations (>100 μM) of farnesol inhibited *S. mutans* growth, consistent with previous observations of its antibacterial effects^39^. However, the presence of *S. mutans* appeared to reduce farnesol production by *C. albicans* (Fig. 6), which may have contributed to hyphal formation as typically observed in co-species biofilms^25^ (Fig. S2). In addition, farnesol can be incorporated into *S. mutans* cell membrane due to its fatty acid-like structure. We have previously shown that farnesol can be detected in the bacterial cell membrane of *S. mutans* biofilm cells^46^ as well as planktonic cells following treatment (Fig. S6). These observations suggest a well-controlled mechanism to maintain farnesol at levels that promote a symbiotic fungal-bacterial relationship, and open intriguing questions as to how cell communication prevent *S. mutans* and *C. albicans* from killing or inhibiting each other.

Nonetheless, the interactions with *S. mutans* in co-species biofilms also provide some advantages to *C. albicans*. The characterization of extracellular sugars and metabolites present in the conditioned media revealed complementary metabolic activity within co-species biofilms. Consistent with the *C. albicans* inability to efficiently metabolize sucrose^29^, the fungus formed sparse biofilms on sHA surface when grown alone with minimal carbohydrate utilization. However, the presence of *S. mutans* and its ability to rapidly breakdown sucrose (releasing glucose and fructose) drastically enhanced *C. albicans* growth within co-species biofilms, accompanied by increased utilization of glucose and fructose. Interestingly, enhanced fructose metabolism can also promote *C. albicans* hyphal morphogenesis^47^,^48^. In line with the metabolic activity of carbohydrates, lactate (primarily) as well as small amounts of formate and fumarate were produced, which, in turn, would favor *S. mutans* and *C. albicans* growth within cariogenic biofilms, as these microbes are both acidogenic and acid-tolerant^49^,^50^. This metabolic cooperation together with modulation of farnesol production provides an effective mechanism that resolves potential antagonism, while promoting co-existence and enhancing *S. mutans* accumulation via Gtf activation.

The findings that *S. mutans* growth stimulation and Gtf activation through bacterial-fungal interactions are achieved within biofilms via secreted molecules also raise new questions. While farnesol at low levels clearly induces bacterial growth and *gtfB* expression simultaneously, the underlying molecular mechanisms are unclear. Recently, Peng *et al*.^51^ have demonstrated that c-di-AMP intracellular signaling in *S. mutans* directly regulates *gtfB* expression. Thus, an intriguing concept may arise where by an extracellular QS molecule from a fungus may trigger intracellular signaling of a bacterial pathogen for enhanced expression of a virulence factor (GtfB) associated with dental caries^7^. Whether other *S. mutans* genes, like *sigX* (which is induced in the presence of *C. albicans*)^31^, are affected or not by farnesol in a dose-dependent manner shall be elucidated by assessing the entire *S. mutans* transcriptome response.

At the same time, the complexity of this bacterial-fungal association exists in many different contexts. Although this study is focused on the influence of the *C. albicans* on *S. mutans* accumulation within biofilms, the interactions between these microbes are mutually beneficial. How *S. mutans* and *C. albicans* interplay within co-species biofilms to promote fungal and bacterial growth while modulating yeast-to-hyphae transition remains unclear as other factors may be involved. For example, *S. mutans*-derived mutanobactin A, competence-stimulating peptide and trans-2-decenoic acid may act as cross-kingdom signaling molecules that regulate *C. albicans* growth and morphogenesis^52^,^53^,^54^. Further analysis on the identity and function of other signaling and small diffusible molecules could reveal a complex and multifaceted role. It is intriguing to think that a highly regulated interplay between cooperative and competitive interactions occurs to allow a symbiotic relationship between these microbes. Thus, it will be interesting to investigate how these different chemical interactors modulate *S. mutans* and/or *C. albicans* physiology using our microcapsule-based nanoculture system. This analytical platform enables the selective diffusion of small molecules while assessing changes in the morphology of the microcolonies, which can help elucidate how community behavior can be modified by sensing secreted molecules under physical confinement^55^,^56^.

In summary, this study provides new insights on the chemical interactions between an opportunistic fungus and an oral pathogen, which in part help to explain the *in vivo* and clinical findings showing enhanced *S. mutans* carriage in the presence of *C. albicans* in plaque-biofilms associated with ECC. Using a microcapsule-based nanoculture system, *S. mutans* cells confined within these chambers responded to chemical interactors (without physical contact between the microbes) that diffused through the PDMS shell. Further chemical analysis identified farnesol as a potential key modulator in this cross-kingdom interaction that, at low concentrations, stimulates bacterial growth and *gtfB* activity, leading to enlarged microcolonies containing densely packed *S. mutans* cells. These observations also suggest new strategies for manipulating this pathogenic bacterial-fungal interaction. For example, high concentration of farnesol can be used to inhibit both *S. mutans* growth and *C. albicans* transition to hyphae, which could lessen the virulence of this cross-kingdom biofilm. In addition, the available evidence prompts the possibility of incorporating antifungal therapy in the treatment of ECC.

## Materials and Methods

### Microbial strains and growth conditions

*Streptococcus mutans* UA159 serotype c (an established virulent cariogenic dental pathogen) and *Candida albicans* SC5314 (a well-characterized fungal strain) were used to generate single- or co-species biofilms. A *S. mutans* strain expressing green fluorescence protein (GFP) under the control of the *gtfB* promoter (P_*gtfB*_::*gfp*) (a gift from Dr. Jose Lemos at College of Dentistry, University of Florida) was used for *in situ gtfB* expression analysis. For inoculum preparation, *C. albicans* and *S. mutans* cells were grown to mid-exponential phase (optical density at 600 nm (OD_600_) of 0.65 and 0.5, respectively) in ultrafiltered (10-kDa molecular-mass cutoff membrane; Millipore, MA, USA) tryptone-yeast extract broth (UFTYE; 2.5% tryptone and 1.5% yeast extract at pH 5.5 and pH 7.0 for *C. albicans* and *S. mutans*) with 1% (wt/vol) glucose at 37 °C and 5% CO_2_ as described previously^17^,^25^.

### Single and co-species biofilm model

Biofilms were formed using our established saliva-coated hydroxyapatite (sHA) disc model as detailed previously^25^ (also see Fig. S1). Briefly, sHA discs were vertically suspended in a 24-well plate using a custom-made disc holder^32^, and inoculated with approximately 2 × 10^6^ (CFU/ml) of *S. mutans* or 2 × 10^4^ (CFU/ml) of *C. albicans* in 2.8 ml (per well) UFTYE (pH 7.0) containing 1% (wt/vol) sucrose at 37 °C under 5% CO_2_. For co-species biofilms, *S. mutans* (2 × 10^6^ CFU/ml) and *C. albicans* (2 × 10^4^ CFU/ml) were added to the inoculum (in 2.8 mL (per well) UFTYE (pH 7.0) containing 1% (wt/vol) sucrose); this proportion of the microorganisms is similar to that found in saliva samples from children with ECC^11^,^25^. The culture medium was changed twice daily at 8 am and 6 pm until the end of the experimental period. The pH of spent culture medium was measured daily at each medium change using a standard pH electrode (Thermo Scientific, Waltham, MA, USA). Representative single or co-species biofilm 3D architecture is shown in Fig. S2.

### Preparation of biofilm-derived conditioned medium

The conditioned medium was collected and prepared from each of the biofilms as detailed in Fig. S1. Briefly, the biofilm and the respective surrounding culture medium were collected at 6, 18 and 30 h, homogenized via sonication and centrifuged at 5,500 × *g* for 10 min at 4 °C. The supernatant was filtered through 0.2 μm-pore-size membrane filter (ultra-low protein binding, surfactant-free cellulose acetate, Nalgene, Rochester, NY, USA), checked for *S. mutans* and *C. albicans* contamination via microscopic observation/plating on blood agar and adjusted to pH 7.0. The cell-free conditioned medium from single-species bacterial (B-CM) or fungal (F-CM) and bacterial-fungal (BF-CM) biofilms were stored at −20 °C.

### Treatments using biofilm-derived conditioned medium

The biological activity of each preparation of the conditioned medium on the growth of *S. mutans* biofilm cells was assessed using the sHA biofilm model^25^,^32^,^33^. Samples of B-CM, F-CM or BF-CM obtained at different time-points (6, 18 and 30 h) were serially diluted with UFTYE (1:2.5, 1:5, 1:10 and 1:20, CM:UFTYE, vol/vol). Thereafter, sucrose was added to each of the culture medium preparations at a final concentration of 1% (wt/vol), and the pH adjusted to 7.0. Each sHA disc was inoculated with 2 × 10^6^ (CFU/ml) of *S. mutans* in 2.8 ml (per well) of UFTYE with or without B/F/BF-CM supplementation, and the biofilm formed at 37 °C under 5% CO_2_. Following 42 h of incubation, the biofilms were collected and analyzed by means of multi-photon confocal microscope and microbiological analysis for 3D biofilm architecture and bacterial viability as described previously^32^,^33^,^57^.

### Quantitative biofilm analysis

The biofilms formed in each condition were examined using confocal microcopy combined with quantitative computational analysis and microbiological assays. Briefly, bacterial cells were stained with 2.5 μM SYTO 9 green-fluorescent nucleic acid stain (485/498 nm; Molecular Probes Inc., Eugene, OR, USA), while EPS was labelled with 1 μM Alexa Fluor 647-dextran conjugate (647/668 nm; Molecular Probes Inc.)^25^,^33^,^57^. The imaging was performed using multi-photon Leica SP5 microscope with 20 × LPlan (numerical aperture, 1.05) water immersion objective (Leica Microsystems, Buffalo Grove, IL, USA). The excitation wavelength was 780 nm, and the emission wavelength filter for SYTO 9 was a 495/540 OlyMPFC1 filter, while the filter for Alexa Fluor 647 was an HQ655/40M-2P filter. The confocal images were analyzed using COMSTAT version 1 (available as free download at http://www.imageanalysis.dk), written as scripts for MATLAB software (Mathworks, Natick, MA, USA) to calculate the biovolume and size (diameter and height) of microcolonies^33^. The biovolume represents the overall biomass occupied by bacterial cells or EPS within intact biofilms, which provide direct measurement of their amounts and ratios across the biofilm depth (from disc surface to fluid phase)^33^. Amira 5.4.1 software (Visage Imaging, San Diego, CA, USA) was used to create 3D renderings to visualize the overall architecture of the biofilms.

Furthermore, a separate set of biofilms was used for standard microbiological analysis. Briefly, the biofilm was removed from sHA discs and homogenized via water bath sonication followed by probe sonication (30 s pulse at an output of 7 W; Branson Sonifier 150, Branson Ultrasonics, Danbury, CT, USA) as described previously^25^,^33^; the sonication procedure does not kill bacterial cells, while providing optimum dispersal and maximum recoverable counts. The homogenized suspension was used to determine the total number of viable cells via plating on blood agar using an automated spiral plater (CFU per biofilm)^39^.

### Glucosyltransferases (Gtfs) activity

The Gtfs activity was measured in each of the conditioned medium and biofilm culture supernatant. An aliquot (100 μl) of the medium (adjusted pH 6.5) was mixed with 100 μl of a [^14^C-glucose]-sucrose substrate (0.2 μCi/ml; 200 mM of sucrose, 40 μM dextran 900, and 0.02% NaN_3_ in buffer consisting of 50 mM KCl, 1 mM CaCl_2_, and 0.1 mM MgCl_2_ at pH 6.5) to a final concentration of 100 mM (200 μl final volume). The reaction mixture was incubated at 37 °C with rocking for 4 h. After incubation, the radio-labeled glucans were quantified by scintillation counting as described elsewhere^58^. Gtfs activity was measured by the incorporation of [^14^C-glucose] from labeled sucrose into glucans. One unit of Gtfs was defined as the amount of enzyme needed to incorporate 1 μM of glucose into glucan over the reaction period^58^.

### Assessment of *gtf* genes expression in biofilms

The dynamics of *S. mutans gtf* genes expression following the exposure of BF-CM (collected at 18 h) was evaluated at specific-time points (see below) over the course of biofilm formation. RNA was extracted and purified using protocols optimized for biofilms formed *in vitro*^59^. Briefly, disc sets were incubated in RNALater (Applied Biosystems/Ambion, Austin, TX, USA) and the biofilm at 18, 22, 28 and 32 h was removed from the sHA discs. The RNAs were purified and DNAse treated on a column using the Qiagen RNeasy Micro kit (Qiagen, Valencia, CA, USA). The RNAs (RNA integrity number of 9.5 or above; as determined using Bioanalyzer, Agilent Technologies Inc., Santa Clara, CA, USA) were then subjected to a second DNaseI treatment with Turbo DNase (Applied Biosystems/Ambion) and purified using the Qiagen RNeasy MinElute Cleanup kit (Qiagen). Then, we performed RT-qPCR to measure the expression profiles of *gtfB* and *gtfC,* which are associated with *S. mutans* microcolony development^33^. Briefly, cDNAs were synthesized using 0.5 μg of purified RNA and the BioRad iScript cDNA synthesis kit (Bio-Rad Laboratories, Inc., Hercules, CA, USA). The resulting cDNAs were amplified with a Bio-Rad CFX96 using previously published specific primers^33^. A standard curve was used to transform the critical threshold cycle (Ct) values to relative numbers of cDNA molecules. Comparative expression was calculated by normalizing each gene of interest to the 16S rRNA^60^.

### Microfluidics-derived nanoculture system

To examine microcolony growth and *in situ gtfB* expression simultaneously, a *gtfB*-green fluorescent promoter strain was encapsulated in a recently developed polydimethylsiloxane (PDMS)-based nanoculture system^34^. Briefly, a glass-capillary microfluidic device with hydrodynamic flow-focusing and co-flowing geometry was constructed to deliver three fluid phases and generate monodisperse water/oil/water (W/O/W) PDMS microemulsion droplets (100–200 μm in diameter), which were allowed to polymerize at 37 °C to form the nanocultures. The three fluid phases were delivered to the microfluidic device through polyethylene tubing (Scientific Commodities, Lake Havasu, AZ, USA) attached to syringes (SGE) that were driven by positive displacement syringe pumps (Harvard Apparatus, Holliston, MA, USA). The inner aqueous phase consisted of defined numbers of *S. mutans* (~30 cells per nanoculture) suspended in UFTYE (a typical low-molecule weight medium) containing 2.5% (wt/vol) sucrose, which was sufficient to reproducibly promote microcolony formation without any influences on the bacterial cell growth. The middle phase consisted of PDMS (Dow Corning Co., Auburn, MI, USA) with 25% (wt/wt) low viscosity silicone oil (50cSt, Thermo Fisher Scientific) and 10% (wt/wt) curing agent. The outside phase comprises 5% (wt/wt) polyvinyl alcohol aqueous solution (PVA, 87–89% hydrolyzed, average MW = 13000–23000, Aldrich). Nanoculture formation was monitored with a high-speed camera (Phantom, Vision Research, Wayne, NJ, USA) attached to an inverted microscope (Eclipse TE200, Nikon, Tokyo, Japan). The nanocultures were collected in a solution of 0.85% NaCl, UFTYE or UFTYE with BF-CM containing 2.5% (wt/vol) sucrose. The *S. mutans* within the nanocultures were grown at 37 °C in the presence of 5% CO_2_ for 24 or 48 h. Images were acquired using confocal laser scanning microscope (SP5-FLIM inverted, Leica Microsystems) or fluorescence microscopy (E600 widefield, Nikon). The fluorescence intensity of GFP was measured using Image J 1.43.

Data are publicly available through the Gulf of Mexico Research Initiative information & Data Cooperative (GRIDC) at http://data.gulfresearchinitiative.org (doi:10.7266/N78G8HQC and doi:10.7266/N7D50K1B).

### Chemical characterization of conditioned medium

The amount of sucrose, glucose and fructose were quantified using high-performance anion-exchange chromatography (HPAEC) equipped with an electrochemical detector (ED50; Dionex, Sunnyvale, CA, USA). A 25 μl portion of aliquot was injected onto a CarboPac PA-1 pellicular anion-exchange column (Dionex), and isocratically separated by 150 mM NaOH (eluent A) at 1 ml/min for 12 min. From the HPAEC chromatograms, the intensities of individual peaks were compared to standard corresponding to glucose, fructose and sucrose, and the peak areas were calculated to determine the amount of respective products. The biofilm-derived metabolites were identified and quantified through ^1^H nuclear magnetic resonance (^1^H-NMR). Briefly, sample (180 μl) was added to 20 μl of D_2_O containing 2,2-Dimethyl-2-silapentane-1-sulfonic acid (DSS, final concentration of 0.25 mM, Cambridge Isotope Limited). All spectra were acquired on a Bruker Avance III HD NMR spectrometer using a triple resonance inverse (TXI) 3 mm probe (Bruker Biospin, Billerica, MA, USA). For high-throughput processing, a Bruker Samplejet was used for sample handling. The pulseprogram (noesypr1d) took the shape of the first transient of a 2 dimensional NOESY of the form RD-90-t-90-tm-90-ACQ (RD = relaxation delay, t = small time delay between pulses, tm = mixing time and ACQ = acquisition). The water signal was saturated using continuous irradiation during RD and tm. The spectra were acquired using 76 K data points over a 14 ppm spectral width. Thirty two scans were performed with 1-s interscan (relaxation) delay and 0.1-s mixing time. Raw free induction decays from the ^1^H-NMR acquisition was processed and profiled using Chenomx NMR suite 8.0 (Chenomx, Alberta, Canada). ^1^H-NMR data was evaluated using targeted profiling strategy^61^ that allows quantification of metabolite data in the sample. The significance was determined by direct comparison with concentration in the blank solution (original UFTYE medium).

### Heat and proteolytic enzyme treatments of the conditioned medium

Each of the conditioned medium (B-CM, F-CM and BF-CM) was subjected to treatments with heat or proteolytic enzyme. Briefly, conditioned medium was adjusted from pH 4.2 to pH 7.5 (as optimum pH of proteinase K). Subsequently pH adjusted conditioned medium was treated with 100 μg/ml proteinase K (Sigma-Aldrich) at 37 °C for 2 h and/or heat at 85 °C for 30 min^62^. The bioactivity (cell growth and Gtfs activity) of each of the treated-conditioned medium was tested as described above.

### Farnesol analysis and bioactivity

To determine whether *C. albicans*-derived farnesol can be detected in the conditioned medium, we used established chromatographic analyses^38^. Farnesol in the conditioned medium was extracted with ethyl acetate with pH adjustment (pH 4) or reverse-phase Sep-Pak column (Mega Bond Elut fresh cartridge, C18, Agilent Technologies, Inc., Santa Clara, CA, USA) without pH adjustment and detected on thin-layer chromatography (TLC) or high performance liquid chromatography (HPLC). Farnesol (*trans, trans*-farnesol) was purchased from Sigma-Aldrich. Farnesol on TLC plates (Slica gel 60F_254_, Merck, Darmstadt, Germany) was identified through *R*_f_ value of standard with detection accomplished with a hand-held UV lamp, iodine vapor and vanillin-sulfuric acid^63^, while farnesol was detected at 196 nm with retention time at 30.5 min using a HPLC system equipped with a Shimadzu LC-20 solvent delivery unit and SPD-M20A photodiode array detector (Shimadzu, Kyoto, Japan). A mobile phase consisted of acetonitrile and H_2_O was delivered under gradient condition on a C18 column (Kinetex C18, 5 μm, 4.6 internal diameter × 250 mm, Phenomenex, Torrance, CA, USA) at a flow rate of 1 ml/min with column temperature set at 40 °C, and injection volume of 20 μl (adapted from Hornby *et al*.^38^). The bioactivity of farnesol on *S. mutans* cell growth, Gtfs activity and microcolony development was performed as described in the previous sections.

### Statistical analysis

Data represent mean ± standard deviations (SD) from at least 3 distinct experiments. The quantitative data were subjected to analysis of variance (ANOVA) in the Tukey’s HSD test for a multiple comparison. A pairwise comparison was conducted using student’s *t*-test.

## Additional Information

**How to cite this article**: Kim, D. *et al*. *Candida albicans* stimulates *Streptococcus mutans* microcolony development via cross-kingdom biofilm-derived metabolites. *Sci. Rep.* **7**, 41332; doi: 10.1038/srep41332 (2017).

**Publisher's note:** Springer Nature remains neutral with regard to jurisdictional claims in published maps and institutional affiliations.

## Supplementary Material

Supplementary Figures
