# Supplementary material for: Candida albicans stimulates Streptococcus mutans microcolony development via cross-kingdom biofilm-derived metabolites
Source: Sci Rep. 2017 Jan 30;7:41332. doi: 10.1038/srep41332 (PMC5278416; doi:10.1038/srep41332)
Supplement: Supplementary Figures [file srep41332-s1.pdf]

Supplementary Information

Candida albicans stimulates *Streptococcus mutans* microcolony development via cross-kingdom biofilm-derived metabolites

Dongyeop Kim[1], Arjun Sengupta[2], Tagbo H. R. Niepa[3], Byung-Hoo Lee[4], Aalim Weljie[2], Veronica S. Freitas-Blanco[5], Ramiro M. Murata[5], Kathleen J. Stebe[3], Daeyeon Lee[3], Hyun Koo[1]

[1]Biofilm Research Laboratory, Department of Orthodontics and Divisions of Pediatric Dentistry & Community Oral Health, School of Dental Medicine, University of Pennsylvania, Philadelphia, PA, USA
[2]Department of Systems Pharmacology & Translational Therapeutics, University of Pennsylvania, Philadelphia, PA, USA
[3]Department of Chemical and Biomolecular Engineering, University of Pennsylvania, Philadelphia, PA, USA
[4]Department of Food Science, Gachon University, Seongnam, South Korea
[5]Herman Ostrow School of Dentistry, University of Southern California, Los Angeles, CA, USA

Figure S1

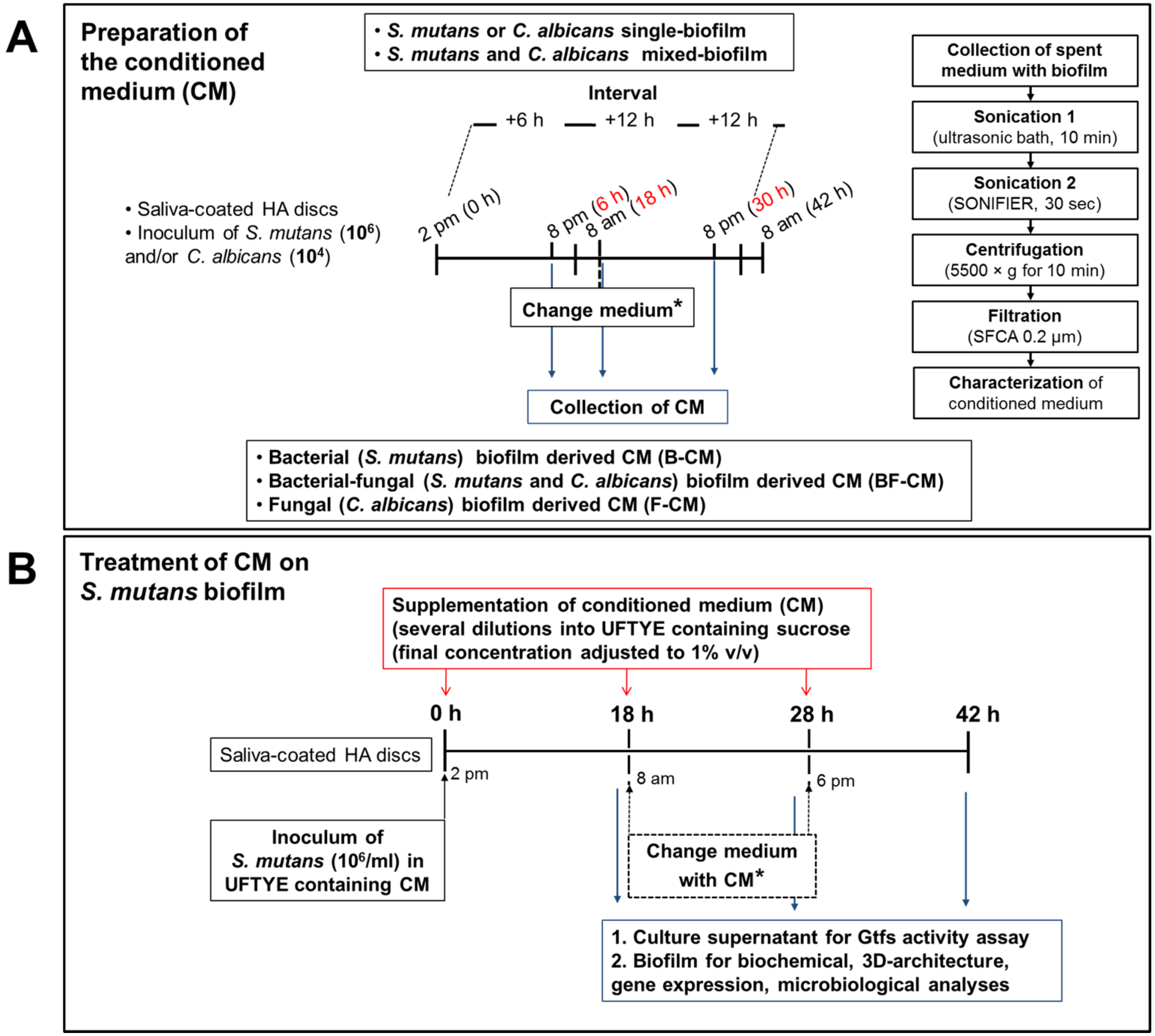

Figure S1. **Experimental design for preparation and treatment of conditioned medium.** Each cell-free conditioned medium (a) was treated to *S. mutans* biofilm (b). *The discs were dip-washed (removal of non-adhered cells) and transferred to fresh culture medium containing 1% sucrose (change medium).

Figure S2

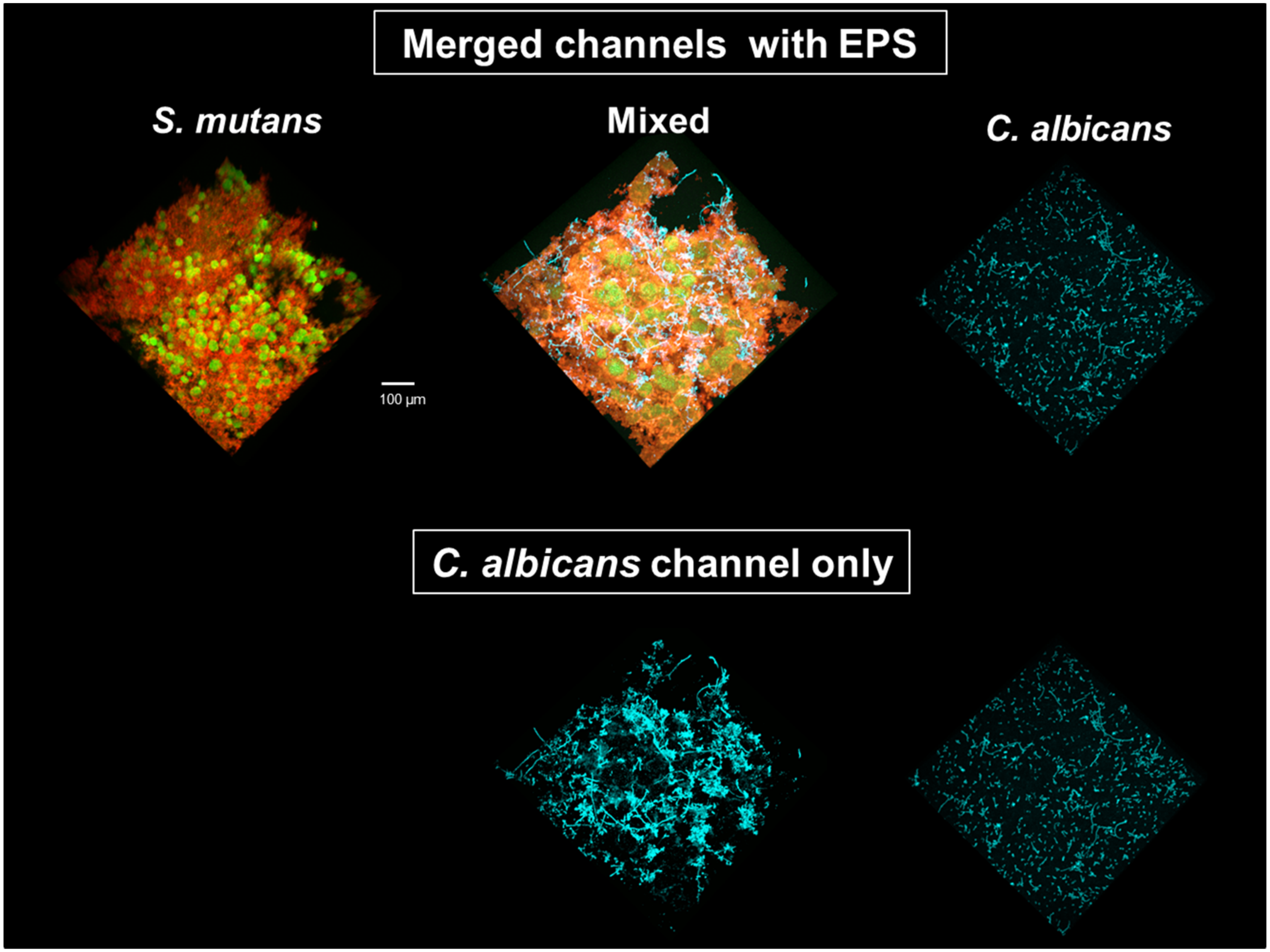

Figure S2. Representative images of single and co-species biofilms grown for 42 h. *S. mutans* cells stained with SYTO 9 are depicted in green, while EPS labelled with Alexa Fluor 647 is in red. *C. albicans* cells stained with Concanavalin A-tetramethylrhodamine appear blue.

Figure S3

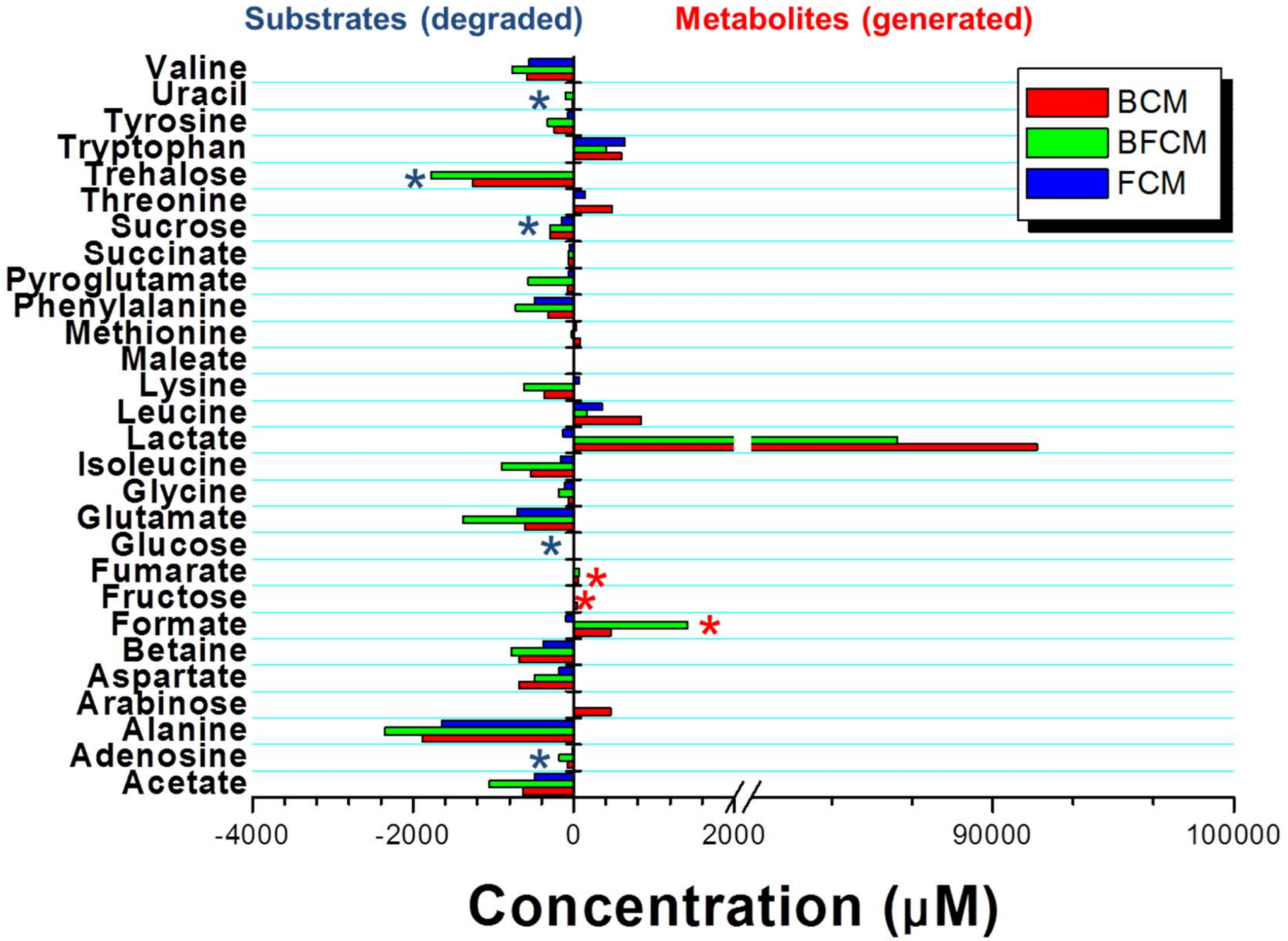

Figure S3. Changes in the concentrations of extracellular metabolites in the conditioned medium (CM). The metabolites were determined by a direct comparison of blank (UFTYE) and CM; higher concentration than the value of blank means metabolites while lower concentration means substrates. The data were subjected to analysis of variance (ANOVA) in the Tukey's HSD test for a multiple comparison (**P*<0.01).

Figure S4

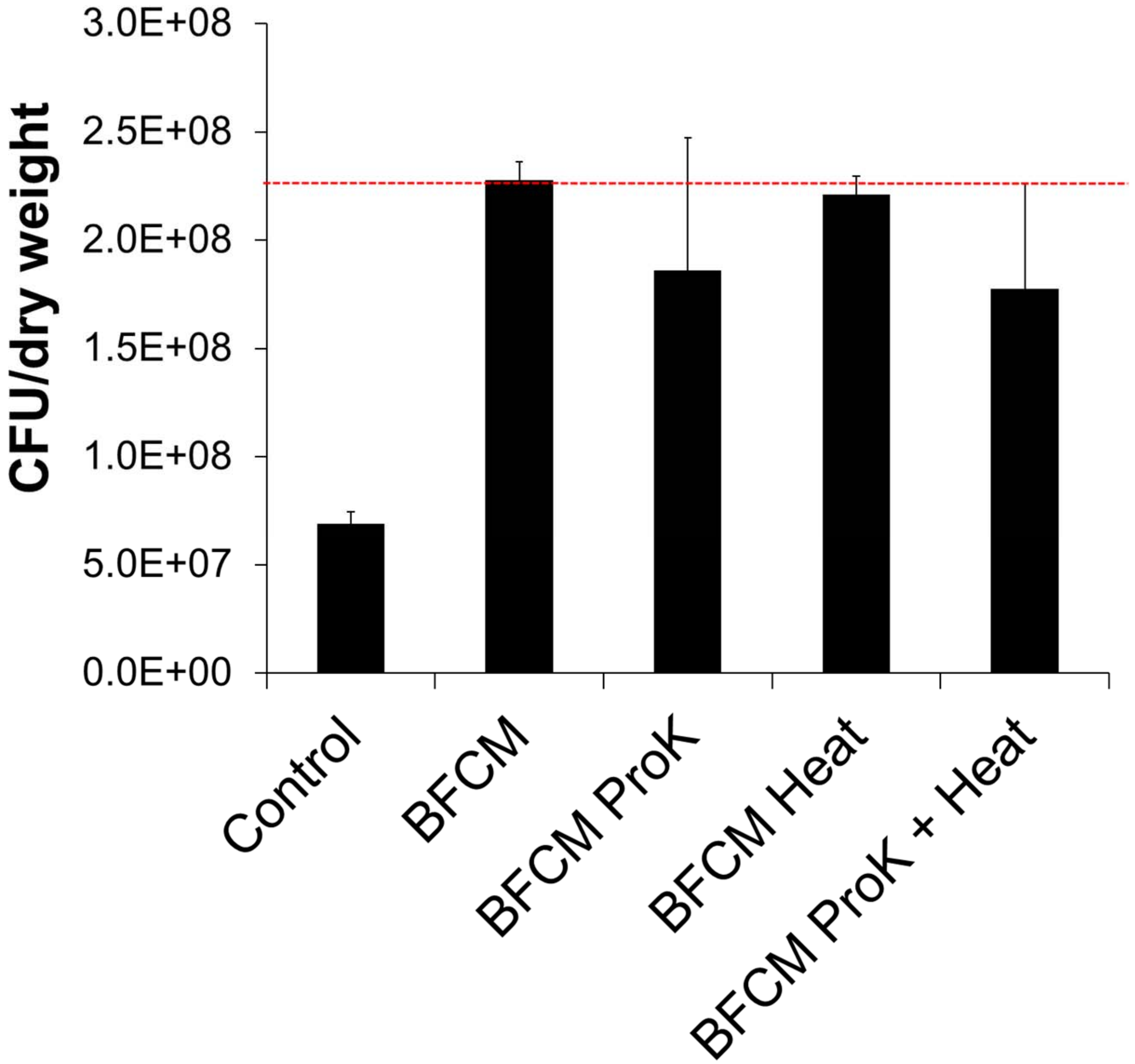

Figure S4. **Physicochemical characterization of bacterial-fungal biofilm derived conditioned medium (BF-CM).** BFCM, original CM; BFCM ProK, proteinase K (100 μg/ml, 37°C for 2 h) treated CM; BFCM Heat, heat (85°C for 0.5) treated CM; BFCM ProK+Heat, proteinase K and heat sequentially treated CM.

Figure S5

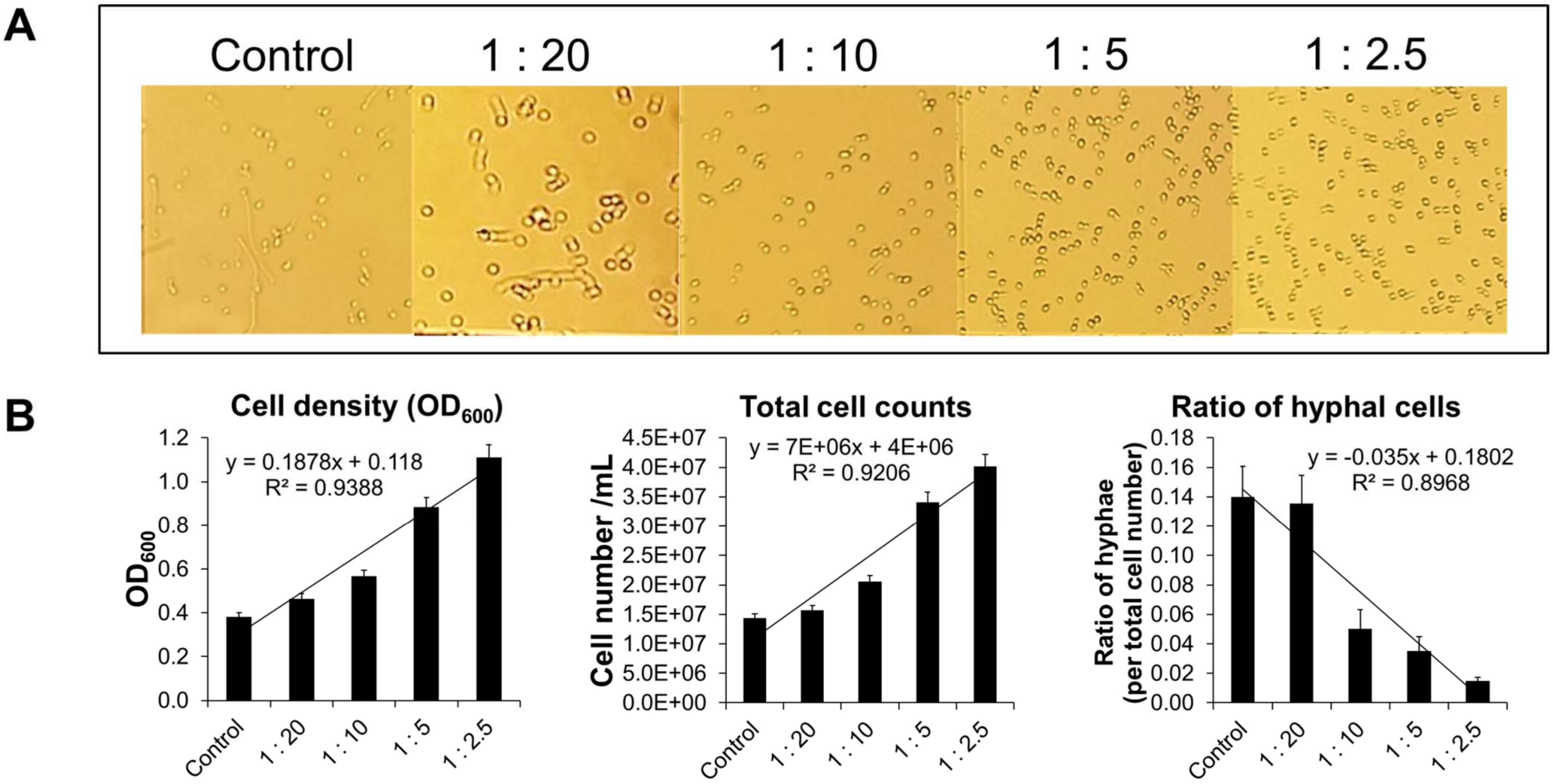

Figure S5. **Morphogenesis of *C. albicans* in the supplementation of BF-CM as a dose-dependent manner.** (A) Influences of BF-CM on morphological change of *C. albicans*. (B) Cell density, total cell number (counted in a hemocytometer) and ratio of hyphal cells were analyzed.

Figure S6

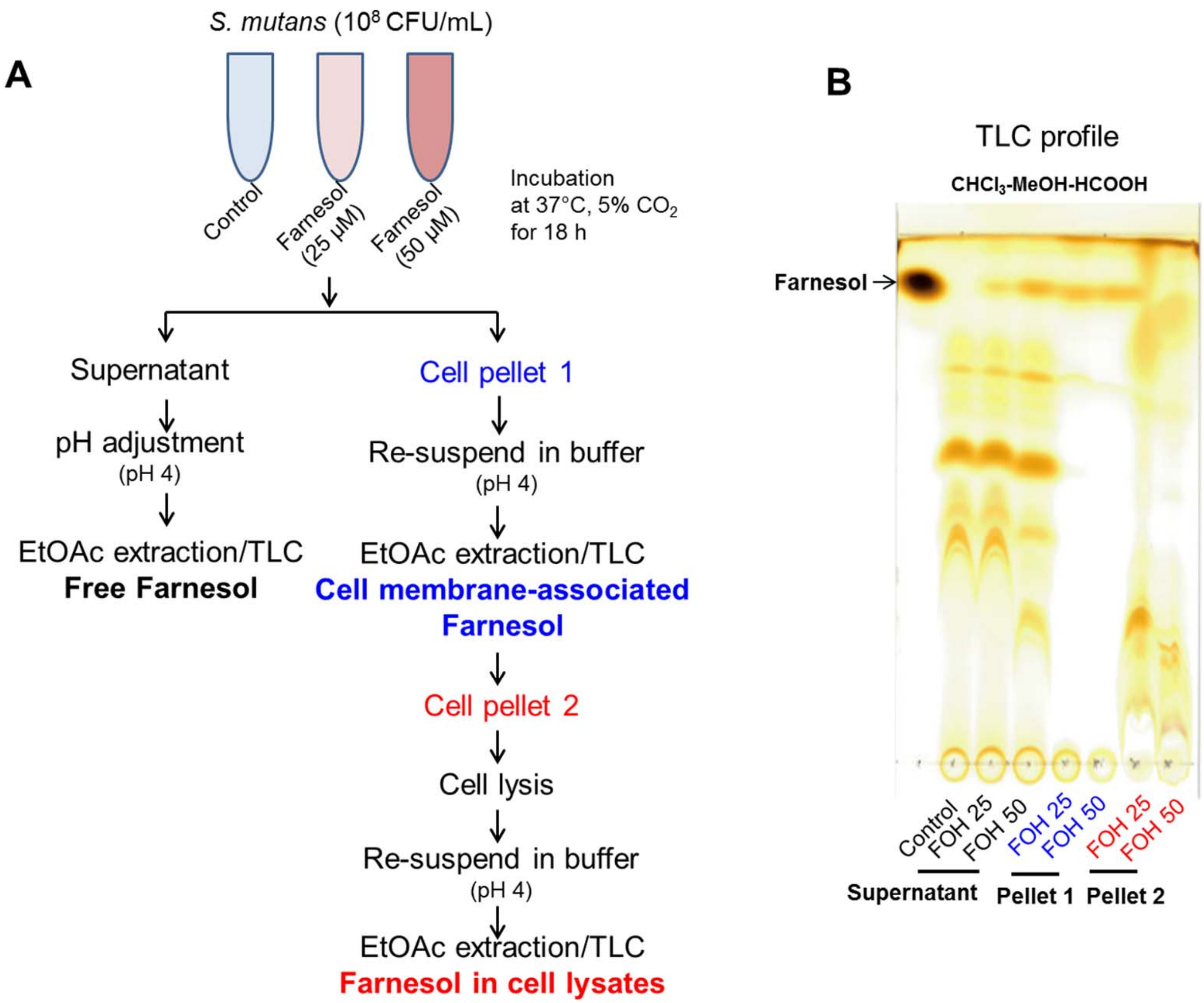

Figure S6. **Detection of farnesol incorporated by *S. mutans*.** (A) Bacterial cell pellets were collected following overnight incubation with farnesol, and then subjected to EtOAc extraction (3 times, 1/5 volume EtOAc) followed by TLC analysis. (B) The EtOAc extracts of cell pellet (Pellet 1; membrane contents were separated into EtOAc layer) contained detectable farnesol, while cell lysates (Pellet 2) showed negligible amounts of farnesol.
